# Mortality rates due to respiratory tract diseases in Tehran, Iran during 2008–2018: a spatiotemporal, cross-sectional study

**DOI:** 10.1186/s12889-020-09495-7

**Published:** 2020-09-17

**Authors:** Elahe Pishgar, Zohre Fanni, Jamileh Tavakkolinia, Alireza Mohammadi, Behzad Kiani, Robert Bergquist

**Affiliations:** 1 Department of Human Geography and Logistics, Faculty of Earth Science, Shahid Beheshti University, Tehran, Iran; 2 Department of Geography and Urban Planning, Faculty of Social Sciences, University of Mohaghegh Ardabili, Ardabil, Iran; 3 Department of Medical Informatics, School of Medicine, Mashhad University of Medical Sciences, Mashhad, Iran; 4 Ingerod, Brastad, Sweden (formerly with the UNICEF/UNDP/World Bank/WHO Special Programme for Research and Training in Tropical Diseases, World Health Organization), Geneva, Switzerland

**Keywords:** Respiratory tract diseases, Mortality, Spatio-temporal analysis, Cluster analysis, Geographical information systems, Tehran, Iran

## Abstract

**Background:**

Tehran, the 22nd most populous city in the world, has the highest mortality rate due to respiratory system diseases (RSDs) in Iran. This study aimed to investigate spatiotemporal patterns of mortality due to these diseases in Tehran between 2008 and 2018.

**Methods:**

We used a dataset available from Tehran Municipality including all cases deceased due RSDs in this city between 2008 and 2018. Global Moran’s *I* was performed to test whether the age-adjusted mortality rates were randomly distributed or had a spatial pattern. Furthermore, Anselin Local Moran’s *I* was conducted to identify potential clusters and outliers.

**Results:**

During the 10-year study, 519,312 people died in Tehran, 43,177 because of RSDs, which corresponds to 831.1 per 10,000 deaths and 5.0 per 10,000 population. The death rate was much higher in men (56.8%) than in women (43.2%) and the highest occurred in the > 65 age group (71.2%). Overall, three diseases dominated the mortality data: respiratory failure (44.2%), pneumonia (15.9%) and lung cancer (10.2%). The rates were significantly higher in the central and southeastern parts of the city and lower in the western areas. It increased during the period 2008–2018 and showed a clustered spatial pattern between 2008 and 2013 but presented a random geographical pattern afterwards.

**Conclusions:**

This study provides a first report of the spatial distribution of mortality due to RSDs in Tehran and shows a significant increase in respiratory disease mortality in the last ten years. Effective control of the excess fatality rates would warrant a combination of urban prevention and treatment strategies including environmental health plans.

## Background

*Respiratory system diseases* (RSDs) represent a major disease burden including death across the world [1, 2]. Although COVID-19 has recently put the respiratory system in focus, the RSDs were leading causes of death and disability already before the advent of this new infection. Indeed, the RSDs stand for five of the thirty most common causes of death worldwide [3, 4]. More than 9.5 million deaths globally are attributed to these diseases every year [5] and four million people die prematurely from chronic respiratory diseases [3]. The most prevalent RSDs are *chronic obstructive pulmonary diseases* (COPD), asthma, acute lower respiratory tract infections, tuberculosis and lung cancer. Their incidence is affected by genetic disposition, gender, lifestyle and age, as well as environmental factors, such as the quality of air and water [4]. In Iran, the *mortality rate due to respiratory system diseases* (MRRSDs) was 9.8% of total deaths in 2019 [6], with the level as high as 14% in the capital city Tehran. This means that 5000 people in Tehran die of RSDs every year, making it one of the most common causes of death in the city from 2008 to 2018 [7]. However, the high MRRSDs is not surprising as Tehran’s population has passed 9 million and the extreme traffic and local industries constantly pollute the air [7]. Since the millennium shift, the municipality has made strong efforts to reduce air pollution by limiting traffic, prohibiting old cars, developing public transport and promoting decentralised residential areas; however, the problem remains despite these initiatives [8]. The high population concentration, the environmental diversity of the surrounding areas (windswept deserts, plains, and mountains) and the presence of many industries [9] have resulted in an extraordinary level of air pollution. There is thus a strong need for an analysis of the current epidemiological status of RSDs in Tehran with special regard to the potential spatial multiplicity of MRRSDs presence.

In recent years, most RSD studies have attempted to examine the association between these diseases and genetic dispositions together with physical activities and socio-economic status in urban environments [10–13]. For example, Apolloni et al. showed that higher assortment of contact patterns strongly increased the probability of spatial containment of the influenza A/H1N1 pandemic; this effect contrasted by an increase in the social activity of adults vs. children [10], while Niyonsenga et al. showed that spatial patterns were inversely related to area-level socio-economic status [11]. They confirmed the importance of individual-level covariates on the prevalence of COPD, especially the number of comorbid conditions as well as age, sex and smoking status [11]. Meanwhile, Adegboye et al. found that interventions to minimise mortality from the Middle East respiratory syndrome (MERS) coronavirus had a particular focus on individuals with co-morbidities, workers outside the health-care field and patients without clinical experience [12], and Chowell et al. noted a high geographic heterogeneity in the impact of the 1918–1919 influenza pandemic mortality in Spain [13]. Other studies have confirmed a significantly robust relationship between MRRSDs and ageing [14–16]. Lai et al. [17] incorporated stochastic processing of environmental and social variables that interact in space and time to affect the patterns of disease transmission in a community. They concluded that some RSDs, such as the severe acute respiratory syndrome (SARS) could potentially have turned into a pandemic helped by environmental determinants and spatial proximity. Their spatial model consistently yielded reliable results for predictions up to 10 days and could therefore aspire to be part of an early warning system (EWS). Other diseases, such as the H1N1 pandemic could change pattern and spread after retrogression and cessation [18].

Another group of previous studies [19, 20] focused on examining the correlation between RSDs and city air quality. In one such study, air pollution was reportedly the cause of 11.2% deaths in the world [21]. Similar studies have been carried out in Tehran, where Mohammadi et al. [22] confirmed increased hospital referrals in December and January of patients with respiratory ailments caused by low temperatures and high-pressure weather conditions leading to inversion and low, dense smoke. Other studies in Tehran [23–28] have found a strong direct relationship between the high density of air gaseous pollutants (O_3_, NO_2_) and micro (μm) particulate matter (PM_2.5_, PM_10_) on the one hand and RSDs on the other. Not surprisingly, smoking is strongly associated with mortality and hospitalisation [26]. Some studies have reported that these pollutants can exacerbate respiratory and pneumonia failure and eventually lead to death [29, 30]. Research shows that genetic factors, smoking, alcohol consumption, radiation exposure and industrial smoke as well as certain occupations and work environment are related to lung cancer [31].

Identification of specific epidemiological patterns helps to implement preventive strategies, which are of special importance in cities where air pollution has a direct impact on RSDs [10, 13]. G*eographical information systems* (GIS) makes it possible to identify spatiotemporal relationships between disease and mortality by combining spatial and temporal data [32], and a few epidemiological studies related to RSDs have been carried out in Iran [33–35]. For example, Sharifi et al. [36] studied risk factors for COPD applying a stratified cluster sampling method in five provinces of Iran, including Tehran. They concluded that less exposure to environmental factors reduces COPD considerably. Yazdani et al. [37] examined tuberculosis (TB) in the province of Mazandaran reporting a heterogeneous spatial pattern regarding the prevalence of this disease. Their findings showed that the TB spatial pattern followed variations in age, sex or population density. Researchers identified hot and cold spots in terms of disease prevalence rate in the study area. Qanbarzadeh et al. [38] investigated the same disease in South Iran, similarly finding clustering of TB cases and the role of spatial factors such as population density. In another but similar study, Khazaei et al. [39] used geographical maps to explore TB prevalence in western Iran, concluding that it had increased by 6.4% between 1992 and 2013, although there were prevalence differences in some regions. Apart from these studies, researchers have been reluctant to apply spatiotemporal methods to analyse RSD data. In particular, the studies have mainly focused on one disease, e.g., influenza [13].

To our knowledge, no study on the spatiotemporal patterns of MRRSDs in Tehran has been done, so we explored this, while also attempting to identify high-risk and low-risk regions in terms of MRRSDs. In addition, since this study was conducted before the COVID-19 pandemic, it provides base data that can be beneficial for future comparison of the effect of COVID-19 pandemic on MRRSDs in Tehran districts.

## Methods

### Study area

**Fig. 1:**
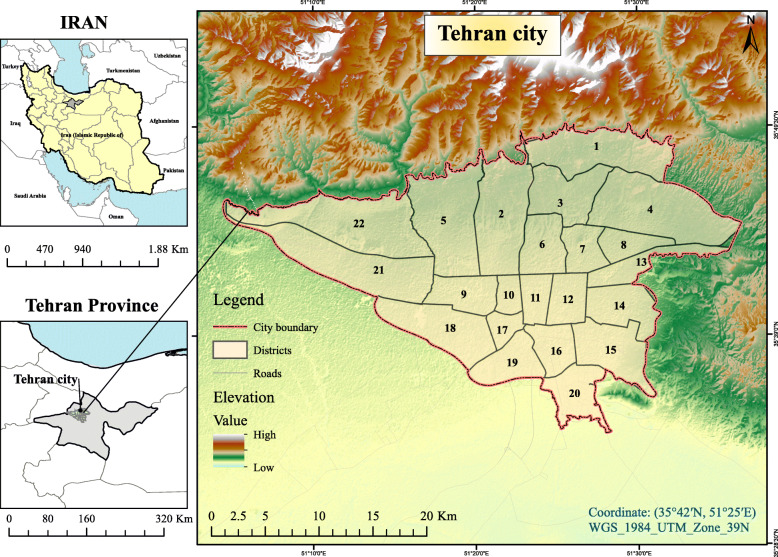
The 22 districts of the city of Tehran. Authors acknowledge the Tehran Municipality for providing the shape file of Tehran districts used to create this map. The shapefile is freely available from https://map.tehran.ir. The elevation data is freely available from https://earthexplorer.usgs.gov. The image was created by ArcGIS 10.7 (ESRI, Redlands, CA, USA)

Tehran, the capital of Iran consists of 22 municipality districts and is located at 35.68.92 N latitude and 51.38.90 E longitude. It is bordered by mountains in the North, plains and urban regions in the West, dry areas in the East and the central deserts of Iran in the South (Fig. 1). According to the latest estimations 2019, the population varies between 8,680,000 at night and around 10,000,000 daytime [40, 41]. It covers an area of 730 km^2^ with a population density of 11,890 per km^2^ [40]. It has a climate with four entirely different seasons [9]. The average annual air temperature in Tehran varies between 15 °C and 18 °C [42].

### Data gathering and preparation

Data on persons deceased due to RSDs from 2008 to 2018 were obtained from the Behesht-e Zahra Organization, a local health department under the supervision of the Tehran Municipality [7]. This dataset includes date of death, age, diagnosis (kind of respiratory disease) and place of residency. Population data were obtained from the statistical centre of Iran [40].

### Calculating age and sex-adjusted MRRSDs

We utilised five age groups, 0–14, 15–24, 25–44, 45–64 and > 65 years, of the census population as basis for our analysis [9]. We calculated the age and sex-adjusted MRRSDs for each district for every year from 2008 to 2018 separately.

### Spatial analysis

There are various methods for measuring spatial autocorrelation. The global methods are more sensitive to departures from the null hypothesis (random distribution) but do not tell where the clusters are, which is possible when applying the local methods [43]. We used *Global Moran’s Index* (GMI) [44, 45] and *Anselin’s Local Moran’s Index* (ALMI) [45, 46] since they, according to the literature [44, 45, 47, 48], are generally more accurate with regard to measuring autocorrelation than other statistics. The radius for the base of proximity analysis was set at 9690.01 m to calculate the GMI and ALMI and the inverse distance method was performed to show spatial relationships. The level of statistical significance was set at *p* = 0.05.

The WGS_1984_UTM_Zone_39N projection system was used for projecting the GIS layers. This projection is a mathematical transformation that transforms spherical coordinates (latitude and longitude) into an XY (planar) coordinate system enabling researchers to create a map that accurately shows distances, areas or directions [49]. WGS_1984_UTM_Zone_39N is suitable for use between 54°E and 60°E, and in the northern hemisphere between the equator and 84°N, onshore and offshore. We used this projection system because the study area is located around 51°E and 35°N.

### Software

Microsoft Excel 2016 and Origin were applied for preparing and encoding the data and drawing diagrams. ArcGIS 10.7 (ESRI, Redlands, CA, USA) was used for performing the spatial statistics. After preparing the initial data, the information was added to the map of Tehran in the ArcGIS environment. The ‘Spatial Statistics Tools’ including ‘Spatial Autocorrelation (GMI)’ and ‘Cluster and Outlier Analysis (ALMI)’ were used to implement the analyses.

## Results

### Descriptive results

**Fig. 2:**
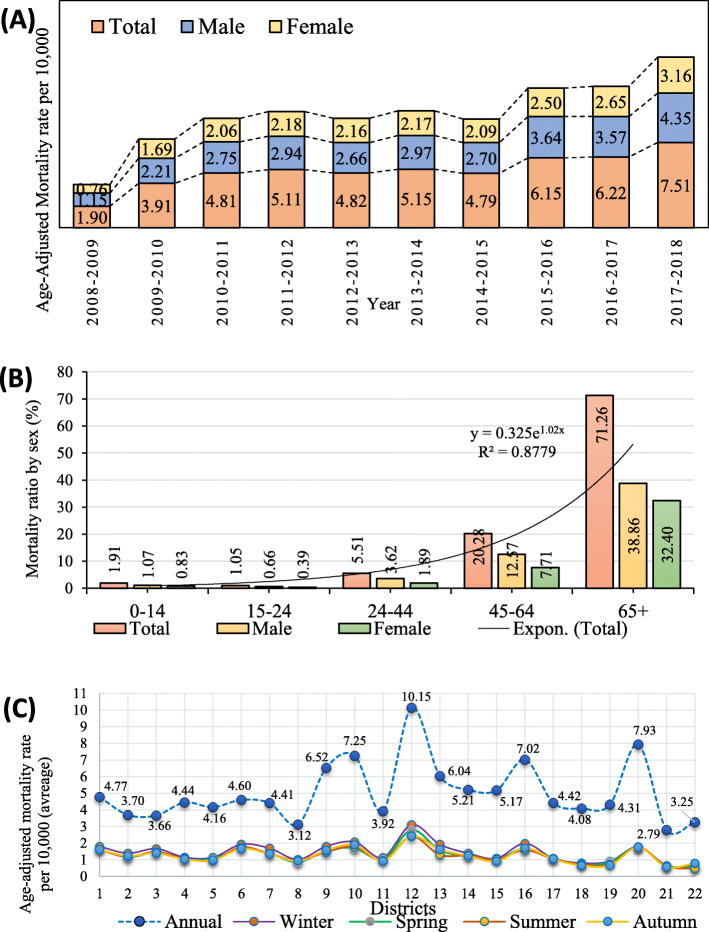
Age-adjusted annual and seasonal mortality rates of respiratory system diseases per 10,000

In the 22 municipality districts of Tehran, a total of 43,177 persons died due to RSDs from 2008 to 2018. During these ten years, the MRRSD average was 5.0 per 10,000, with men being at higher risk of death than women (56.8% versus 43.2%). The average of 1.9 per 10,000 in 2008 had quadrupled to 7.5 per 10,000 deaths in 2018. Figure 2-A reveals a continually ascending trend of MRRSDs over the ten years of the study, particularly after 2015 (4.8 per 10,000). The mean MRRSDs was 2.9 for males and 2.1 for females (Fig. 2-a). Fig. 2-b shows the MRRSDs separately by gender and age groups, indicating that 71.2% of the deaths occurred in the age group > 65 years. Figure 2-c, depicting the MRRSDs by municipality district, reveals that district 12 had the highest rate and district 21 the lowest. As seen in Fig. 1, district 12 is located in the centre of Tehran (downtown) and district 21 in the western part of the city.

Figure 2-c reveals that district 12 encountered higher seasonal MRRSDs compared to the other areas during all four seasons throughout the time covered by the study. District 21 and 22 that are located in the western part of the city had the lowest seasonal mortality rates. Most seasonal rates were observed during winter and autumn. Furthermore, the highest rates were often observed in the central and southeastern regions, while those located in the West encountered low rates (Fig. 4).

### Cause of mortality

**Fig. 3:**
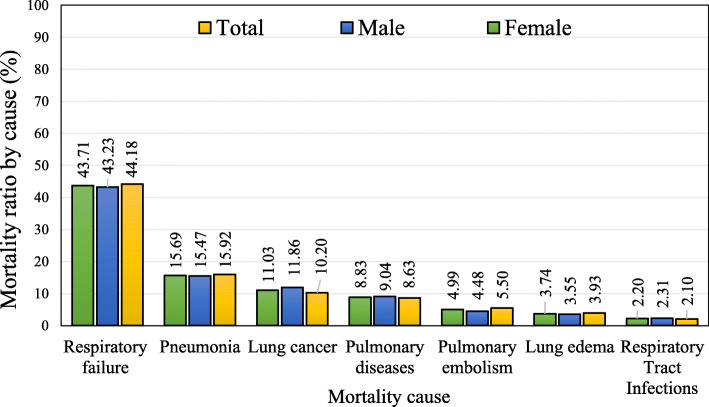
Respiratory system diseases caused deaths by sex, Tehran, 2008–2018

Thirty-four leading causes of death were related to RSDs in the data obtained from Tehran Municipality. Out of these causes, the following MRRSD groups dominated (91.2%) the picture in Tehran: respiratory failure (44.2%), pneumonia (15.9%), lung cancer (10.2%), pulmonary diseases (exact type not given) (8.6%), pulmonary embolism (5.5%), lung oedema (3.9%) and respiratory infections (2.1%) (Fig. 3).

### Spatiotemporal results

#### Annual rates

**Fig. 4:**
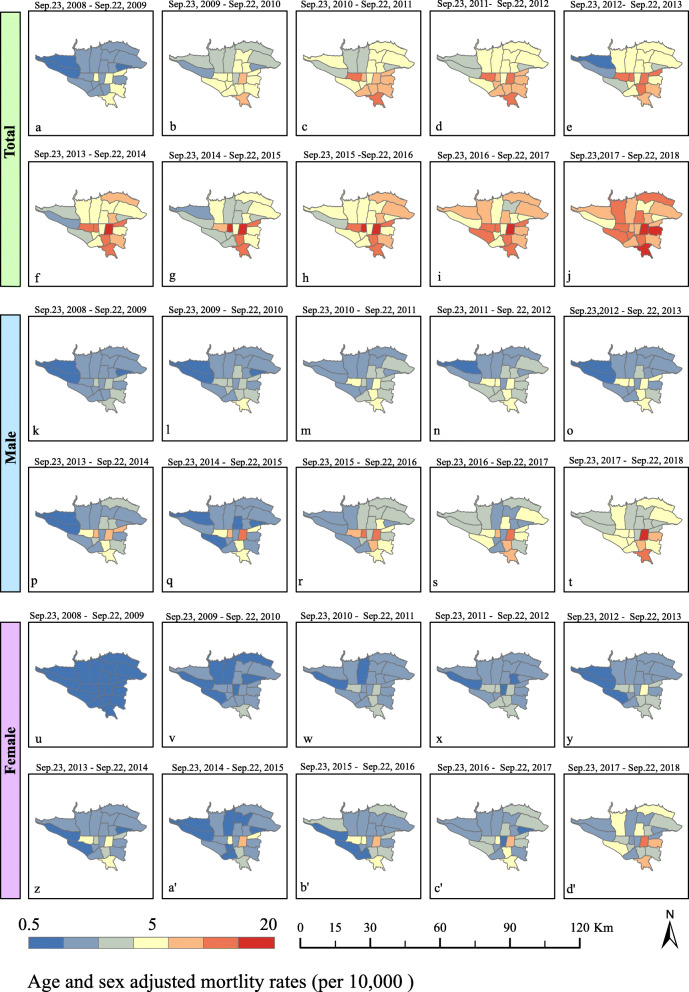
Annual age-adjusted MRRSDs by sex in different districts of Tehran 2008–2018. Authors acknowledge the Tehran Municipality for providing the shape file of Tehran districts used to create this map. The shapefile is freely available from https://map.tehran.ir. The image was created by ArcGIS 10.7 (ESRI, Redlands, CA, USA)

Figure 4 shows the age-adjusted MRRSDs from 2008 to 2018 for both genders in the 22 municipality districts of Tehran. It is obvious that the total number of age-adjusted MRRSDs has increased considerably from 2008 to 2018; for instance, the overall mortality in district 12 (downtown) increased from 2.9 per 10,000 to 19.3. Moreover, the overall geographical distribution of MRRSDs also increased over the ten-year study period from 5 to 19 high-rate districts over the study period.

Compared to other areas, the rate of age-adjusted MRRSDs was considerably less in the western parts of Tehran. Some of the southern and central parts of the city encountered high rates throughout the study. Figure 4(k-t) shows that the male mortality rates were considerably different from both the female ones and the total. According to Fig. 4(u-d’), at the end of the period in 2018 and among all regions, the maximum rate of age-adjusted MRRSDs was not higher than 7.7 per 10,000 for women, which is in contrast to the male mortality at 11.6 and the total at 10.1.

#### Spatial autocorrelation

Table 1 shows the results of applying GMI for the age-adjusted MRRSDs per 10,000 from 2008 to 2018. The dominant pattern of spatial autocorrelation was that of the high clustering (HC) from 2008 to 2010. However, there was low clustering (LC) during 2010–2013 (*P < 0*.07); therefore, the null hypothesis (lack of spatial autocorrelation) was rejected for the period 2008–2012. The spatial distribution pattern changed gradually from 2013 to 2018, eventually confirming the null hypothesis. In other words, random spatial autocorrelation was observed in this period (*P* > 0.1). Performing analysis by gender showed that MRRSDs in females was only high-clustered in two of the years (Table 1).

**Table 1:** Results of Global Moran’s *I* analysis of mortality rate due to respiratory system diseases in Tehran, 2008–2018

Year (***Sept. 23 to Sept. 22***)	Moran’s ***I***			Critical value			Stat. significance			Variance			Expected index			Spatial distribution pattern		
				(*Z*-score)			(***P***-value)											
	*Tot.*	*Male*	*Fem.*	*Tot.*	*Male*	*Fem.*	*Tot.*	*Male*	*Fem.*	*Tot.*	*Male*	*Fem.*	*Tot.*	*Male*	*Fem.*	*Tot.*	*Male*	*Fem.*
2008–2009	.509	.328	.468	4.180	2.942	3.895	.00	.00	.00	.017	.016	.017	−.048	−.048	−.048	HC	HC	HC
2009–2010	.363	.387	.155	3.137	3.293	1.560	.00	.00	.11	.017	.017	.016	−.048	−.048	−.048	HC	HC	R
2010–2011	.192	.272	.029	1.797	2.369	.587	.07	.01	.55	.017	.018	.016	−.048	−.048	−.048	LC	LC	R
2011–2012	.281	.336	.130	2.467	2.904	1.324	.01	.00	.18	.017	.017	.018	−.048	−.048	−.048	LC	HC	R
2012–2013	.260	.179	.333	2.309	1.693	2.902	.02	.09	.00	.017	.017	.017	−.048	−.048	−.048	LC	LC	HC
2013–2014	.115	.168	.000	1.255	1.656	0.371	.20	.09	.07	.017	.017	.016	−.048	−.048	−.048	R	LC	R
2014–2015	.087	.074	.092	1.074	.966	1.129	.28	.33	.26	.016	.015	.015	−.048	−.048	−.048	R	R	R
2015–2016	.039	.053	.009	.669	.078	.445	.50	.43	.65	.017	.016	.016	−.048	−.048	−.048	R	R	R
2016–2017	−.030	.006	−.060	.148	3.37	−.107	.88	.73	.91	.013	.014	.014	−.048	−.048	−.048	R	R	R
2017–2018	−.019	.017	−.069	.251	.566	−.183	.80	.57	.85	.013	.013	.014	−.048	−.048	−.048	R	R	R

Abbreviations: ***C*** clustered, ***R*** random, *L* low, ***H*** high

#### Cluster and outlier analysis

**Fig. 5:**
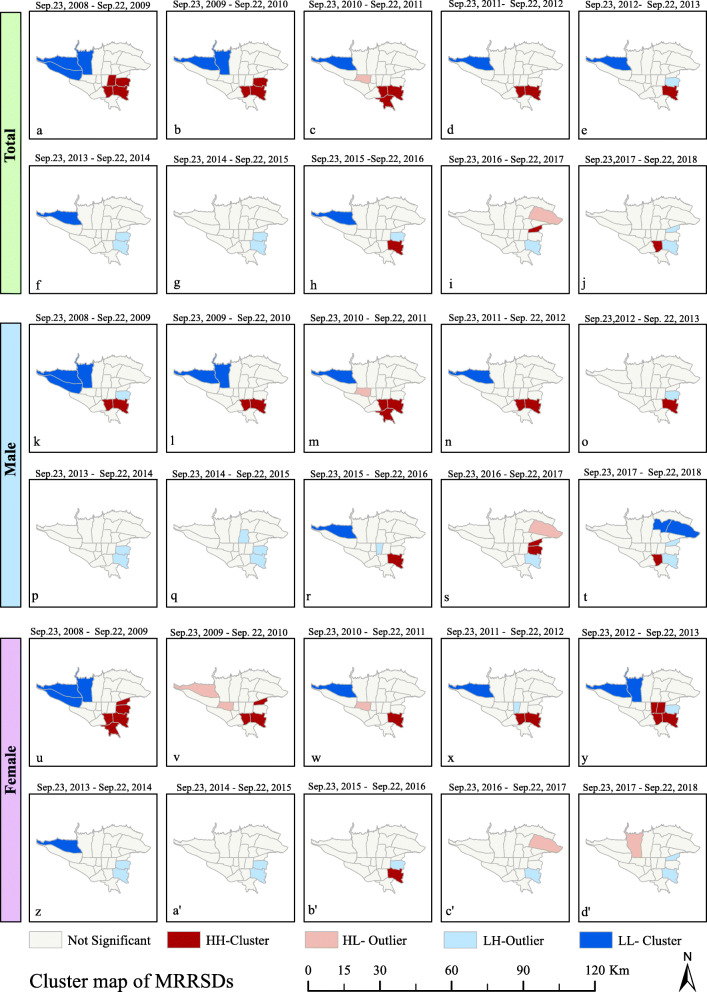
Age-adjusted cluster map according to Anselin Local Moran’s *I* of MRRSDs in Tehran 2008–2018. Authors acknowledge the Tehran Municipality for providing the shape file of Tehran districts used to create this map. The shapefile is freely available from https://map.tehran.ir. The image was created by ArcGIS 10.7 (ESRI, Redlands, CA, USA)

Figure 5 shows the results of ALMI in terms of the total annual age-adjusted MRRSDs. Districts 15 and 16 exhibited a *high-high* (HH) cluster from 2008 to 2013, while some regions, e.g., district 11 showed a *low-high* (LH) cluster from 2008 to 2018 and district 22 was in a *low-low* (LL) cluster (*P* < 0.05) at that time. ALMI results by sex followed that of the total age-adjusted MRRSDs with few changes. It faded gradually from 2014 to 2018 and then turned into a random pattern. It means that MRRSDs have reached other regions of Tehran in recent years. Thus, despite the increase in deaths during the period, for reasons not covered in this study, MRRSDs have spread to other districts but with no MRRSD clusters in recent years.

## Discussion

Respiratory failure (44.2%), pneumonia (15.9%), and lung cancer (10.2%) were found to be the leading causes of death among respiratory patients in our study. This is expected due to the highly polluted environment of Tehran, specifically in relation to presence of increased levels of NO_2_ and PM_2.5_ [8]. These pollutants can intensify respiratory failures and pneumonia, leading to death in the long term [28, 50]. In addition, social-economic factors and immobility predispose for an increased incidence of COPD [51, 52]. Since most providers of cigarettes and water pipes are more common in the southeastern parts of the city than other municipalities [40], these results are in line with previous studies confirming that smoking in Tehran increases the incidence of lung cancer [26]. Rohani- Rasaf et al. [53] concluded that the death rate from cancers, including lung cancer, has increased over time in Tehran’s lower socio-economic districts. Furthermore, previous studies [26, 53] have shown that lung cancer is responsible for increasing of death rates, especially in the elderly in some districts (e.g. district 12); as in this study, those areas also had high mortality rates.

Our results show that age-adjusted MRRSDs almost quadrupled from 2008 to 2018 but was slightly lower in 2012 and 2013. The cause(s) of this increase are not known but could include increased air pollution, smoking, overcrowding and other potential environmental risk factors. Our results also show that the rate of age-adjusted MRRSDs was higher in men than women, which is in line with the results by Yu et al. [16], who reported an even more significant difference (81.2% versus 18.2%) in Changzhou, China. This is not surprising as men are supposedly more exposed to environmental factors than women [23]. The age-adjusted MRRSDs were higher in the > 65 years age group, which has also been reported in various geographical regions [14–16].

It was noted that the central and southeastern parts of Tehran had the highest rate of MRRSDs (Fig. 4) and also most businesses and official buildings are situated in these areas; For instance, the high-MRRSD district 12 includes not only crowded driveways but also has the highest number of gas stations [54, 55]. The western parts of Tehran, characterised by a relatively low population density and fewer buildings, had the lowest MRRSDs rate. Furthermore, this area is exposed to dominant winds, is geographically elevated and has natural air ducts [9]. The southern areas, in contrast, is the site of big industrial workshops and the amount of air pollution is higher in southern parts [40], which can be an explanation for the high MRRSDs rate found in this part of the city.

We also saw the MRRSDs gradually and significantly increase from 2008 to 2018; for instance, the total MRRSDs went from 2.8 per 10,000 in the western parts to 10.1 in the central parts. These results are in line with those of other researchers [56], who state that the increase in mortality is due to the high number of RSDs in Tehran. The results of analysing the seasonal rates pattern showed that high rates in some parts of the city (District 12 in downtown) and low rates in the other parts (district 21 in the West) repeated year by year along the season changes in winter and autumn. Inversion happens in autumn along with the educational centres’ activity and gradual temperature drop; therefore, the highest air pollution generally appears in the autumn [41]. Having examined Tehran records, it was obvious that the air pollution index was commonly higher during the autumn-winter period from 2013 to 2018 [57]. As some researchers have reported, MRRSDs can significantly increase during the cold season [16, 17, 19–22].). In addition to air pollution, increased respiratory deaths in winter is likely due to increased respiratory infections. Another possible cause of seasonal differences could be temperature (increased deaths with cold temperature). It should be noted that all of these reasons presented in this study are hypotheses that should be considered in future studies.

### Recommendations

An association with of elevated values of certain factors leading to RSDs mortality in megacities has been shown by this study and others. It is now up to the authorities to create and enforce policies leading to long-term reduction of these factors. It is recommended to combine social policies like decreasing tobacco usage with urban planning. Giving license to pollutant businesses should be limited in some regions like central and southeastern districts that now have the highest MRRSDs. Examining and promoting safety and health in working environments, especially in regions with high mortality rates in men can help to decrease the development of RSDs and overall mortality. Clean public transports infrastructure (electric cars) and removal of factories from the southeastern parts of Tehran would probably assist prevention of respiratory diseases there.

### Limitations

Mortality statistics of smaller urban scales were not available. Therefore, we chose urban districts as the analytical basis.

## Conclusions

Our findings indicate a significant increase in respiratory disease mortality in Tehran in the last 10 years. Furthermore, the spatiotemporal distribution of MRRSDs in Tehran follows a heterogenic pattern after 2013. Considering effective control of MRRSDs would warrant a combination of prevention and treatment strategies through urban planning including environmental pollutant control and environmental health plans. This should be taken into consideration by health policymakers when facing the increasing risk of mortality in relation to environmental factors.

## Data Availability

Raw data on persons deceased due to RSDs from 2008 to 2018 were obtained from the Behesht-e Zahra Organization, a local health department under the supervision of the Tehran Municipality. There is no permission to obtain the datasets and they are available from the corresponding author on request.

## Abbreviations

RSDs: Respiratory system diseases
COPD: Chronic obstructive pulmonary diseases
MRRSDs: `Mortality rates due to respiratory system diseases
SARS: Sever acute respiratory syndrome
GIS: Geographical information systems
GMI: Global Moran’s Index
ALMI: Anselin’s local Moran Index
LISA: Local indicators of spatial autocorrelation
HC: High-clustered
LH: Low-clustered
